# Arenobufagin induces MCF-7 cell apoptosis by promoting JNK-mediated multisite phosphorylation of Yes-associated protein

**DOI:** 10.1186/s12935-018-0706-9

**Published:** 2018-12-18

**Authors:** Li-Juan Deng, Ming Qi, Qun-Long Peng, Min-Feng Chen, Qi Qi, Jia-Yan Zhang, Nan Yao, Mao-Hua Huang, Xiao-Bo Li, Yin-Hui Peng, Jun-Shan Liu, Deng-Rui Fu, Jia-Xu Chen, Wen-Cai Ye, Dong-Mei Zhang

**Affiliations:** 1 Guangdong Province Key Laboratory of Pharmacodynamic Constituents of Traditional Chinese Medicine and New Drugs Research, Jinan University, Guangzhou, 510632 China; 2 Formula-pattern Research Center, School of Traditional Chinese Medicine, Jinan University, Guangzhou, 510632 People’s Republic of China; 3 College of Pharmacy, Jinan University, Guangzhou, 510632 People’s Republic of China; 4 Department of Pharmacology, School of Medicine, Jinan University, Guangzhou, 510632 People’s Republic of China; 5 School of Traditional Chinese Medicine, Southern Medical University, Guangzhou, 510515 People’s Republic of China; 6 Guangzhou Yucai Middle School, Fujin Road 2#, Dongshan District, Guangzhou, China

**Keywords:** Arenobufagin, YAP, JNK, Breast cancer, Apoptosis, Bufadienolide

## Abstract

**Background:**

It has been demonstrated that bufadienolides exert potent anti-cancer activity in various tumor types. However, the mechanisms that underlie their anti-cancer properties remain unclear. Yes-associated protein, a key effector of Hippo signaling, functions as a transcription coactivator, plays oncogenic and tumor suppressor roles under different conditions. Here, we report that arenobufagin (ABF), a representative bufadienolide, induced breast cancer MCF-7 cells to undergo apoptosis, which occurred through the JNK-mediated multisite phosphorylation of YAP.

**Methods:**

Cytotoxicity was examined using an MTT assay. ABF-induced apoptosis was measured with a TUNEL assay and Annexin V-FITC/PI double staining assay. Western blotting, immunofluorescence, qRT-PCR and coimmunoprecipitation were employed to assess the expression levels of the indicated molecules. Lose-of-function experiments were carried out with siRNA transfection and pharmacological inhibitors. ABF-induced phosphopeptides were enriched with Ti4^+^-IMAC chromatography and further subjected to reverse-phase nano-LC–MS/MS analysis.

**Results:**

ABF significantly reduced the viability of MCF-7 cells and increased the percentage of early and late apoptotic cells in a concentration- and time-dependent manner. Following ABF treatment, YAP accumulated in the nucleus and bound to p73, which enhanced the transcription of the pro-apoptotic genes *Bax* and *p53AIP1*. YAP knock-down significantly attenuated ABF-induced apoptotic cell death. Importantly, we found that the mobility shift of YAP was derived from its phosphorylation at multiple sites, including Tyr357. Moreover, mass spectrometry analysis identified 19 potential phosphorylation sites in YAP, with a distribution of 14 phosphoserine and 5 phosphothreonine residues. Furthermore, we found that the JNK inhibitor SP600125 completely diminished the mobility shift of YAP and its phosphorylation at Tyr357, the binding of YAP and p73, the transcription of *Bax* and *p53AIP1* as well as the apoptosis induced by ABF. These data indicate that ABF induced YAP multisite phosphorylation, which was associated with p73 binding, and that apoptosis was mediated by the JNK signaling pathway.

**Conclusions:**

Our data demonstrate that ABF suppresses MCF-7 breast cancer proliferation by triggering the pro-apoptotic activity of YAP, which is mediated by JNK signaling-induced YAP multisite phosphorylation as well as its association with p73. The present work not only provides additional information on the use of ABF as an anti-breast cancer drug, but also offers evidence that the induction of the tumor suppressor role of YAP may be a therapeutic strategy.

**Electronic supplementary material:**

The online version of this article (10.1186/s12935-018-0706-9) contains supplementary material, which is available to authorized users.

## Background

Breast cancer is the most common malignant tumor in women, as approximately 630 thousand deaths and 2.1 million newly diagnosed cases occurred worldwide in 2018, which accounted for almost 25% of all cancer cases among women [1]. Although current treatments for breast cancer, such as surgery, endocrine therapeutics, and radiation therapy are used [2, 3], metastasis and acquired and/or intrinsic endocrine resistance remain the greatest clinical challenges in breast cancer treatment [3–6]. Therefore, chemotherapeutic agents with new mechanisms that induce breast cancer cell death are urgently needed.

Yes-associated protein (YAP), one of the Hippo signaling pathway effectors, functions as a key node of multiple signaling pathways and plays multiple roles by interacting with various transcription factors under different stimulants [7, 8]. As a transcriptional coactivator, YAP lacks DNA-binding domain and must interact with DNA-binding transcription factors to initiate downstream gene expression. To activate the transcription of genes involved in cell proliferation and apoptosis inhibition, YAP mainly binds to TEAD family members [9, 10]. However, YAP also functions as an apoptosis inducer, as evidenced by the finding that YAP binds to DNA-binding tumor suppressors, including RUNXs and p73 [11]. Thus, YAP can act either as an oncogene or a tumor suppressor, which is primarily dependent on its particular binding partners. Several studies have reported that phosphorylation modification is critical for the interaction between YAP and its binding partners, as these modifications regulate its stability, activity, and intracellular trafficking [7]. The phosphorylation of YAP at Ser94 promotes interaction between YAP and TEAD, which is required for YAP-induced cell proliferation [12, 13]. However, Ser127 and Ser381, which are the principal phosphorylation sites of LATS1/2, induce YAP to be sequestered in the cytoplasm, where it can undergo degradation; this results in the suppression of its oncogenic activity [14]. In response to DNA damage, YAP is phosphorylated at Tyr357 by c-Abl, which increases protein stability and its binding affinity with p73. This leads to elevated expression of p73-mediated pro-apoptotic target genes [15]. Considering these findings, the phosphorylation events of YAP provide potential therapeutic targets, and small molecules that regulate YAP phosphorylation can be utilized as a treatment option for cancer.

Bufadienolides such as arenobufagin (ABF), bufalin, bufotalin and cinobufagin are the main active ingredients of Chan’su and Cinobufacini (Huachansu injection), which are used alone or in combination with other chemotherapeutic drugs to clinically treat various cancers in China and East/Southeast Asian countries [16–18]. Moreover, many studies have revealed their broad-spectrum antitumor activities in vitro and in vivo in cancers such as liver cancer, lung cancer and colon cancer [18–20]. Previously, we found that ABF possessed potent antitumor activity in several human cancer cell lines, which was accompanied by the following: induction of apoptosis via PI3 K/Akt signaling [20] and the ClC-3 chloride channel [21], disruption of the cell cycle through the ATM/ATR pathway [22], suppression of epithelial-to-mesenchymal transition (EMT) through the Wnt/β-catenin pathway [23–25], and inhibition of angiogenesis mediated by VEGFR-2 signaling [26]. However, little attention has been dedicated to its anti-breast cancer effects as well as the underlying mechanisms.

In the current study, we present the effects of ABF on human breast cancer cell line MCF-7. The data show that ABF induces apoptosis in human breast cancer MCF-7 cells, which is triggered by YAP multisite phosphorylation and interaction with p73, and that it is mediated by JNK signaling. These results not only provide a theoretical basis for the clinical use of ABF as an anti-breast cancer reagent, but also offer evidence for inducing YAP apoptotic function as a therapeutic strategy.

## Materials and methods

### Reagents and antibodies

ABF (purity > 98%) was purchased from Baoji Herbest Bio-Tech Co., Ltd. (Baoji, Shanxi, China). 4′,6-Diamidino-2-phenylindole (DAPI) and 3-(4,5-dimethylthiazol-2-yl)-2,5-diphenyltetrazolium bromide (MTT) were supplied by Sigma-Aldrich (St. Louis, MO, USA). An in situ cell death detection kit was obtained from Roche (Indianapolis, IN, USA). Antibodies against caspase-9 (#9504), cleaved caspase-9 (#9509), PARP (#9542), cleaved PARP (#5625), YAP (#14074), p-YAP (Ser127) (13008), LATS1 (#9153), p38 (#8690), p-p38 (#4511), ERK1/2 (#9102), p-ERK1/2 (#8544), MEK (#2352), p-MEK (#26975), JNK (#9252), p-JNK (#4668), β-actin (#3700), Ki67 (#9449), rabbit IgG (#7074) and mouse IgG (#7076) were obtained from Cell Signaling Technology (Beverly, MA, USA). The antibody against p-YAP (Tyr357) (ab62751) was obtained from Abcam (Cambridge, MA, USA). SB203580 (a p38 MAPK inhibitor), SP600125 (a JNK inhibitor) and U0126 (a MEK1/2 inhibitor) were purchased from Selleck Chemicals (Houston, Texas, USA). An E.Z.N.A.^®^ total RNA kit was obtained from Omega (Norcross, Georgia, USA). SuperZyme III cDNA Synthesis SuperMix and Biotool™ 2× SYBR Green qPCR Master Mix were supplied by Bimake (Shanghai, China). An Annexin V-FITC and PI apoptosis kit was purchased from Life Technologies (New York, USA). Pierce™ protein G magnetic beads and a BCA protein assay kit were obtained from Thermo Fisher Scientific (Waltham, MA, USA). An in situ cell death detection kit was purchased from Roche Diagnostics (Basel, Switzerland). Alkaline phosphatase was obtained from Beyotime Biotechnology (Guangzhou, Guangdong, China).

### Cell lines and cell culture

The human breast cancer cell line MCF-7 was obtained from the Chinese Academy of Sciences Cell Bank (Shanghai, China). Cells were cultured in DMEM containing 1% antibiotics (penicillin and streptomycin) and 10% fetal bovine serum and were maintained at 37 °C in a humidified atmosphere of 5% CO_2_. The MCF-7 cell line used in this study was identified by short tandem repeat (STR) profiling and was not contaminated by mycoplasma detected by using TransDetect PCR^®^ Mycoplasma Detection assay kit.

### Cell viability assay

The cytotoxicity of ABF in MCF-7 cells was assessed using an MTT assay. Cells (5 × 10^3^ cells/well) were plated on 96-well plates and cultured for 24 h. Cells were incubated with various concentrations of ABF for 24 h, 36 h, 48 h or 72 h. Then, cell viabilities were tested by the addition of MTT to determine the generation of formazan crystals in the presence of viable cells. Following 4 h of incubation at 37 °C, the purple dye was dissolved in 100 μL of DMSO. The absorbance was detected by a microplate reader at 570 nm. Cells treated with 0.2% DMSO in medium were considered 100% viable.

### Annexin V-FITC/PI double staining assay

ABF-induced apoptosis in MCF-7 cells was quantified by an Annexin V-FITC/PI double staining assay kit. After exposure to ABF in the presence or absence of siRNA, YAP (100 nM) or SP600125 (10 μM), MCF-7 cells were harvested and resuspended in 500 μL of dyes containing Annexin V-FITC and PI for 15 min in the dark. The cells were analyzed by flow cytometry at an excitation wavelength of 488 nm and an emission wavelength of 525 nm. The total number of apoptotic cells was calculated from the PI^−^/Annexin V^+^ region plus the PI^+^/Annexin V^+^ region of the raw flow cytometry plot, as previously reported [27, 28].

### TUNEL assay

DNA fragmentation was examined using a TUNEL assay. MCF-7 cells (5 × 10^3^ cells/well) were plated on 6-well plates and cultured for 24 h. The cells were then incubated with various concentrations of ABF for 12 h, 24 h, or 36 h. Then, the cells were fixed in 4% paraformaldehyde (PFA) and permeabilized with 0.1% Triton X-100. After incubation with the TUNEL reaction mixture, the cells were stained with DAPI in the dark and observed by fluorescence microscopy.

### Western blotting analysis

Cells were lysed in buffer (25 mM Tris–HCl, pH = 7.6, 150 mM NaCl, 1% NP-40, 1% sodium deoxycholate, 500 mM DTT, 100 mM PMSF and 0.1% SDS), and the lysate was collected. Total protein concentration was determined using a BCA protein assay kit. Proteins (40 μg) were separated by SDS-PAGE and then transferred to polyvinylidene (PVDF) membranes. The membranes were blocked and subsequently immunoblotted with antibodies against the target proteins overnight at 4 °C. The membranes were incubated with secondary antibodies for 1 h at room temperature. Enhanced-chemiluminescence substrates were employed to visualize the immunoreactive bands using an X-ray film processor. Quantitative results for the shifting of YAP were defined as follows: Shift ratio of YAP (%) = *a*/*b* × 100%, where *a* = the band intensity of YAP shift part, *b* = the band intensity of YAP whole, this was evaluated using ImageJ Software.

### Immunofluorescence

Cells treated with ABF were fixed in 4% formaldehyde, permeabilized with 1% Triton X-100, and blocked with 5% BSA. Then, the cells were incubated with primary antibodies for 2 h at room temperature and then stained with Alexa Fluor 488-conjugated secondary antibodies and DAPI dye. Images were acquired using a laser scanning confocal microscope (LSM800, Zeiss).

### Coimmunoprecipitation

The coimmunoprecipitation procedure using Pierce™ protein G magnetic beads was performed as previously described [22]. Cells were harvested and resuspended in pre-chilled lysis buffer supplemented with protease and phosphatase inhibitors. Samples were lysed on ice for 30 min, and then, the cell lysates were collected by centrifugation at 15,000*g*, 10 min at 4 °C. Protein concentrations were determined with a BCA assay. One milligram of protein extract was incubated with an antibody against YAP or YAP (Tyr357) overnight at 4 °C after incubation with G magnetic beads for 2 h. The immunoprecipitated complex was washed, centrifuged and dissolved in 2× loading buffer. The samples were then analyzed by SDS polyacrylamide gel electrophoresis and immunoblotting, as described above.

### Quantitative real-time PCR

Total RNA was isolated from MCF-7 cells using an E.Z.N.A. Total RNA kit according to the manufacturer’s instructions. First-strand cDNA was produced from 500 ng of total RNA using SuperZyme III cDNA Synthesis SuperMix. Real-time PCR was performed with a Biotool™ 2× SYBR Green qPCR Master Mix kit in a 20 µL reaction mixture containing 25 ng of cDNA. The sequence-specific oligonucleotide primers are listed in Additional file 1: Table S1. The relative mRNA expression levels of the target genes were calculated using the 2^−ΔΔCT^ method, and the fold-change expression normalized to the control was calculated for all samples.

### siRNA transfection

Cells were transfected with the target siRNA duplexes or control siRNA using Lipofectamine 3000 according to the manufacturer’s protocol. The target siRNA duplexes are listed in Additional file 1: Table S2. After 48 h of incubation, cell lysates were harvested and target proteins were examined by Western blotting. The transfected cells were exposed to ABF, which was followed by an analysis of cell viability and apoptosis.

### Enrichment of phosphopeptides by Ti4^+^-IMAC chromatography

The phosphopeptide enrichment procedures using Ti4^+^-IMAC beads were performed as previously described [29]. The total peptide mixture was resuspended in DHB buffer (80% CAN, 3% DHB and 0.1% TFA) with Ti4^+^-IMAC bead slurry for 30 min at 4 °C. After incubation, the supernatant was removed by centrifugation and the beads were washed three times with 500 μL washing solution I (50% ACN, 6% TFA, and 200 mM NaCl) and 500 μL washing solution II (30% ACN, 0.1% TFA) each time. Subsequently, the bound phosphopeptides were eluted with 200 μL of 10% v/v NH_3_·H_2_O. The supernatant was collected, desalted with C18 Cartridge (66872-U, Sigma), and lyophilized until dry.

### LC–MS analysis

The lyophilized peptides were reconstituted in 20 μL of 0.1% FA and 6 μL of the sample was analyzed by an online reverse-phase LC–MS/MS system consisting of an Eksigent NanoLCUltra 2D plus system (AB SCIEX) coupled with a Q Exactive mass spectrometer (Thermo Fisher Scientific, CA, USA) via a nanoelectrospray source. Peptide mixtures were loaded onto a reverse-phase trap column (100 μm × 2 cm, nanoViper C18, Thermo Scientific Acclaim PepMap100) connected to the C18 reversed-phase analytical column (75 μm × 10 cm, 3 μm, Thermo Scientific Easy Column) in buffer A (0.1% FA) and separated with a linear gradient of buffer B (84% ACN and 0.1% FA) at a flow rate of 300 nL/min. The Q Exactive spectrometer was operated in positive ion mode, and survey scan MS spectra (from m/z 300–1800) were acquired at a resolution of 70,000. System control and data collection were performed by MASCOT 2.2 (Matrix Science).

### Statistical analysis

For the statistical analysis, GraphPad Prism 5.0 was used to perform a one-way ANOVA with Tukey’s test or a two-tailed unpaired *t* test. *P* < 0.05 was regarded as statistically significant.

## Results

### ABF causes MCF-7 cell death through the induction of apoptosis in vitro

**Fig. 1:**
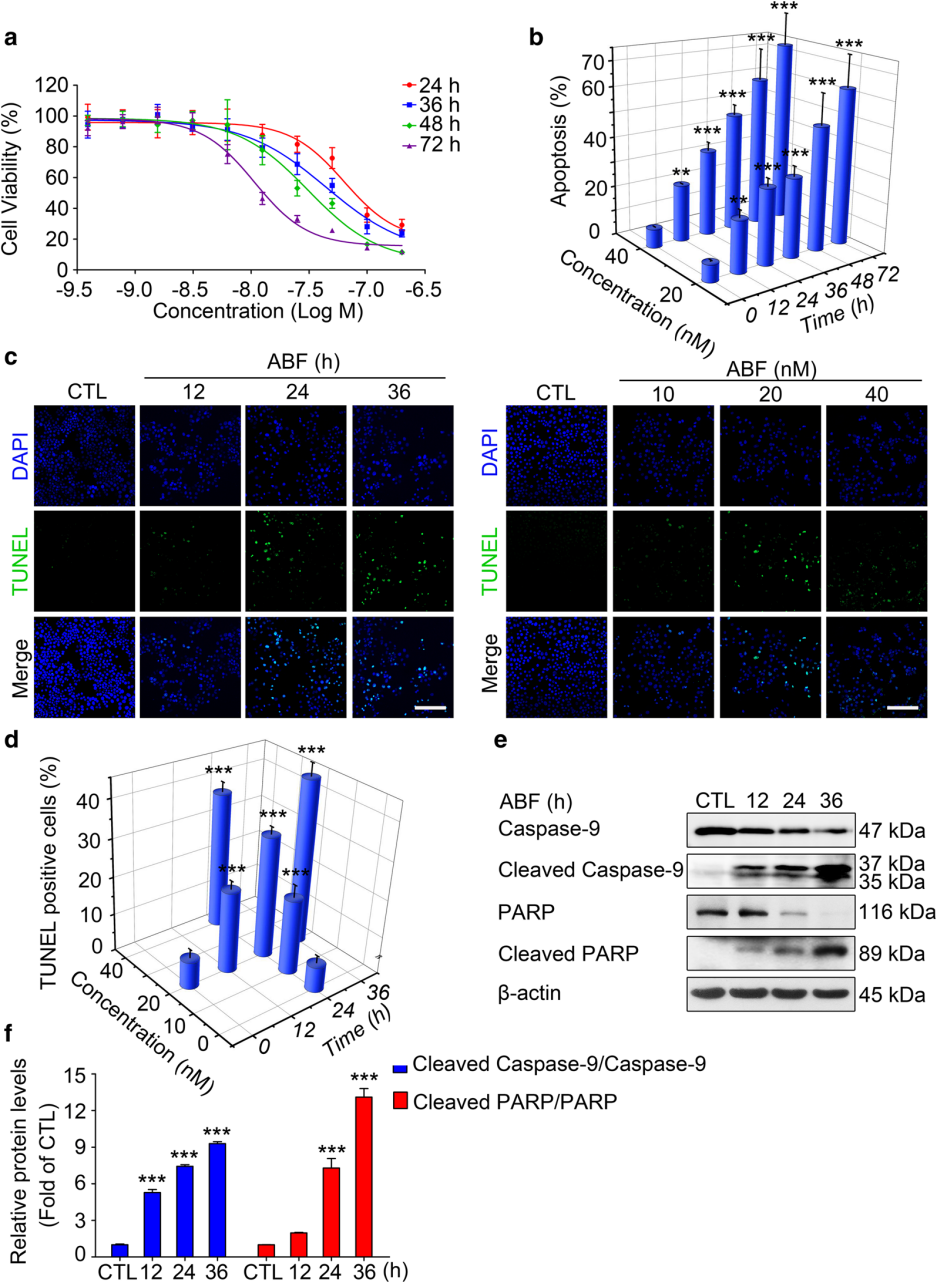
Anticancer activity of ABF in MCF-7 cells in vitro. **a** The concentration–response curves of ABF in MCF-7 cells. Cell viability of MCF-7 cells treated with different concentrations of ABF for 24 h, 36 h, 48 h or 72 h was measured using an MTT assay. Data are shown as the mean ± SEM (n = 3). **b** ABF induces apoptosis in a time- and concentration-dependent manner. Following treatment, cell apoptosis was analyzed by Annexin V-FITC/PI staining assay. Data represents the mean ± SEM (n = 3). ^**^P < 0.01, and ^***^P < 0.001 versus the control. **c** Apoptotic effect of ABF was determined by a TUNEL assay. TUNEL (green) was used to mark fragmented DNA. DAPI (blue) was used to indicate the cell nuclei. Original magnification: 200× ; Scale bar: 200 μm. **d** The quantitative data of the TUNEL assay. Data represent the mean ± SEM, n = 3. ^***^P < 0.001 versus the control. **e** Effects of ABF on the expression of apoptosis-related proteins. Whole-cell lysates extracted from MCF-7 cells treated with ABF (20 nM) were evaluated by Western blotting. β-actin was used as the loading control. **f** Quantitative data of the protein levels described above. Data represent the mean ± SEM, n = 3. ^***^P < 0.001 versus the control

The in vitro anticancer effect of ABF on MCF-7 cells was investigated by MTT assay. ABF reduced the viability of MCF-7 cells in a concentration- and time-dependent manner, with IC_50_ values of 80.23 ± 2.25 nM, 49.96 ± 0.13 nM, 23.00 ± 0.27 and 11.07 ± 0.29 nM following treatment with ABF for 24 h, 36 h, 48 h and 72 h, respectively (Fig. 1a). Apoptosis in ABF-treated cells was analyzed using the Annexin V/PI staining assay. ABF treatment significantly increased the percentage of early and late apoptotic cells in a concentration- and time-dependent manner (Fig. 1b and Additional file 1: Fig. S1). DNA fragmentation was observed in cells treated with ABF for 12 h, 24 h and 36 h by the TUNEL assay, which suggests the occurrence of cell apoptosis (Fig. 1c, d). In addition, apoptosis-associated proteins, including cleaved caspase-9 and cleaved PARP, were clearly upregulated by ABF treatment (Fig. 1e, f). Taken together, these results indicate that ABF reduces MCF-7 cell proliferation in vitro through the induction of apoptosis.

### Involvement of YAP in ABF-induced apoptosis

**Fig. 2:**
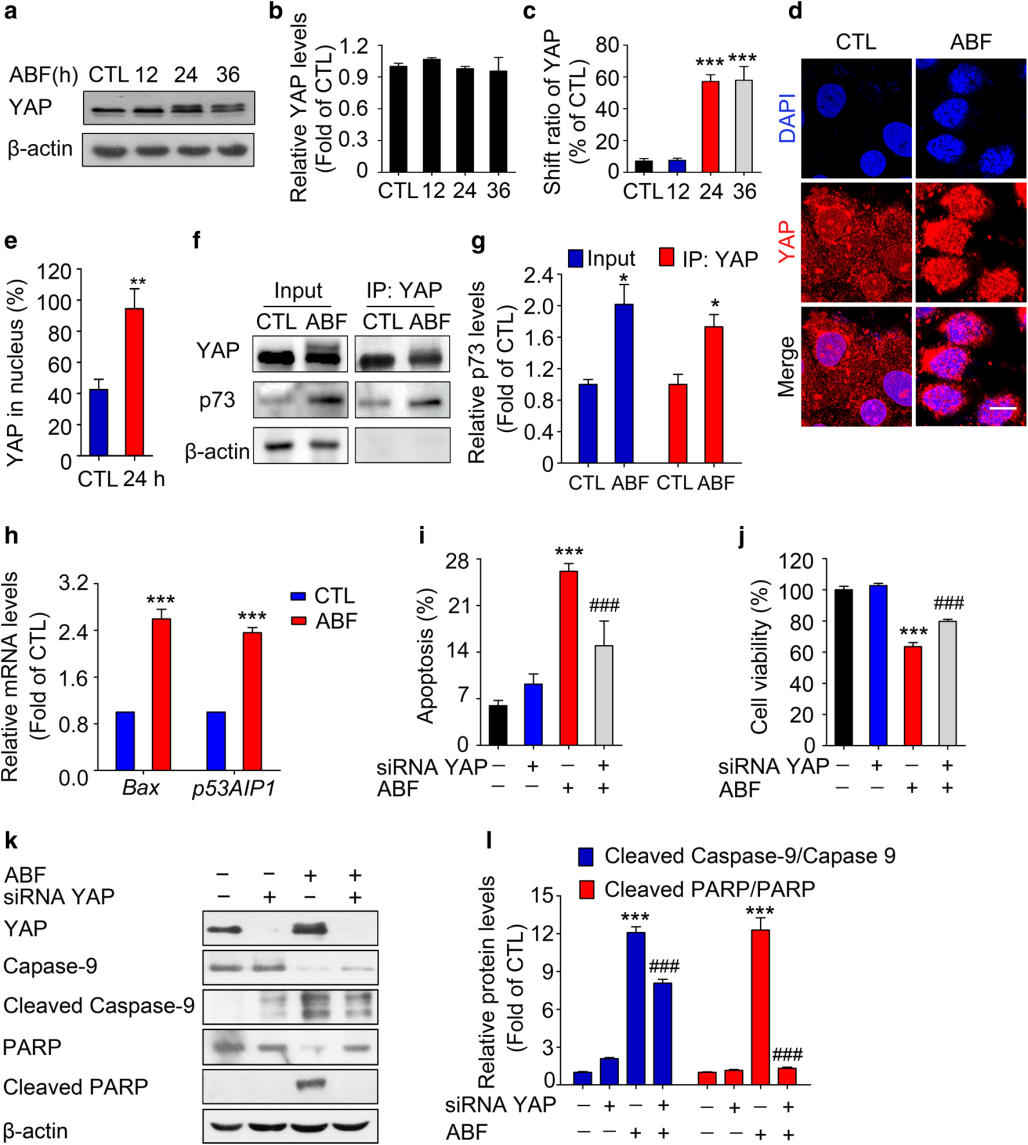
Involvement of YAP in ABF-induced apoptosis. **a** Effects of ABF (20 nM) on the expression of YAP in MCF-7 cells. Whole cell lysates were evaluated by Western blotting with β-actin as the loading control. **b** Quantification of protein levels was analyzed by ImageJ Software. Data represent the mean ± SEM, n = 3. ^***^P < 0.001 versus the control. **c** Quantitative data of the YAP shift induced by ABF. Data represent the mean ± SEM, n = 3, ^***^P < 0.001 versus the control. **d** ABF promotes the nuclear translocation of YAP. Following treatment with ABF (20 nM), the cells were fixed and incubated with the indicated antibodies. Immunofluorescence images were obtained by confocal scanning laser microscopy (LSM 800, Zeiss). Original magnification: 400 × ; Scale bar: 10 μm. **e** Quantitative results of the relative fluorescence intensity of YAP in the nucleuses. Data represent the mean ± SEM, n = 5, ^**^P < 0.01 versus the control. **f** The level of the YAP/p73 complex in ABF-treated cells. The YAP/p73 complex was pulled down by coimmunoprecipitation with an anti-YAP antibody. Total cell lysates were used as the input control. **g** Quantification of the YAP/p73 complex in ABF-treated cells. Data represent the mean ± SEM, n = 3, ^*^P < 0.05 versus the control. **h** ABF (20 nM) upregulated the mRNA levels of *Bax* and *p53AIP1*. The mRNA levels of *Bax* and *p53AIP1* were determined by quantitative real-time PCR. Each column represents the mean ± SEM (n = 3). ^***^P < 0.001 versus the control. **i**–**l** YAP siRNA antagonizes ABF-induced apoptosis. Cells were pretreated with YAP siRNA and incubated with ABF (20 nM). Then, treated cells or cell lysates were evaluated by the Annexin V-FITC/PI staining assay (**i**), cell viability assay (**j**), and Western blotting (**k**). **l** Quantification of the expression of the indicated proteins. Each column represents the mean ± SEM, n = 3. ^***^P < 0.001 versus the control; ^###^*P* < 0.001 versus the ABF group

When cells are exposed to apoptotic conditions, YAP is an important determinant of the progression of apoptosis [30]. Here, our data showed that the protein levels of YAP in ABF-treated cells were unchanged, but shifts in the mobility of YAP were notable in cells treated with ABF for 24 h and 36 h (Fig. 2a–c). As a crucial regulator, YAP is located in the nucleus where it binds to a series of pro-apoptotic transcription factors, such as p73, to enhance the expression of pro-apoptotic genes, such as *Bax* and *p53AIP1*, which subsequently induces apoptosis [15, 31, 32]. Therefore, we next examined the subcellular distribution of YAP in ABF-treated cells. In untreated cells, YAP localized evenly in both the cytoplasm and the nucleus. YAP localized selectively in the nuclei of cells treated with ABF for 24 h (Fig. 2d, e). We further examined the YAP/p73 complex. As shown in Fig. 2f, g, ABF treatment resulted in a notable increase in the YAP/p73 complex. Furthermore, the pro-apoptotic factors *Bax* and *p53AIP1* were upregulated at the mRNA level in ABF-treated cells (Fig. 2h). These results reveal that YAP is potentially associated with ABF-induced apoptosis. Thus, this action of YAP was investigated by siRNA silencing. The combined treatment with ABF and YAP siRNA significantly reduced the apoptotic cell population and cell viability compared with ABF treatment alone (Fig. 2i, j). The upregulation of cleaved caspase-9 and cleaved PARP was dramatically attenuated by YAP siRNA pretreatment (Fig. 2k, l). Collectively, these data demonstrate that YAP plays an important role in ABF-induced apoptosis.

### ABF induces YAP phosphorylation at multiple sites

**Fig. 3:**
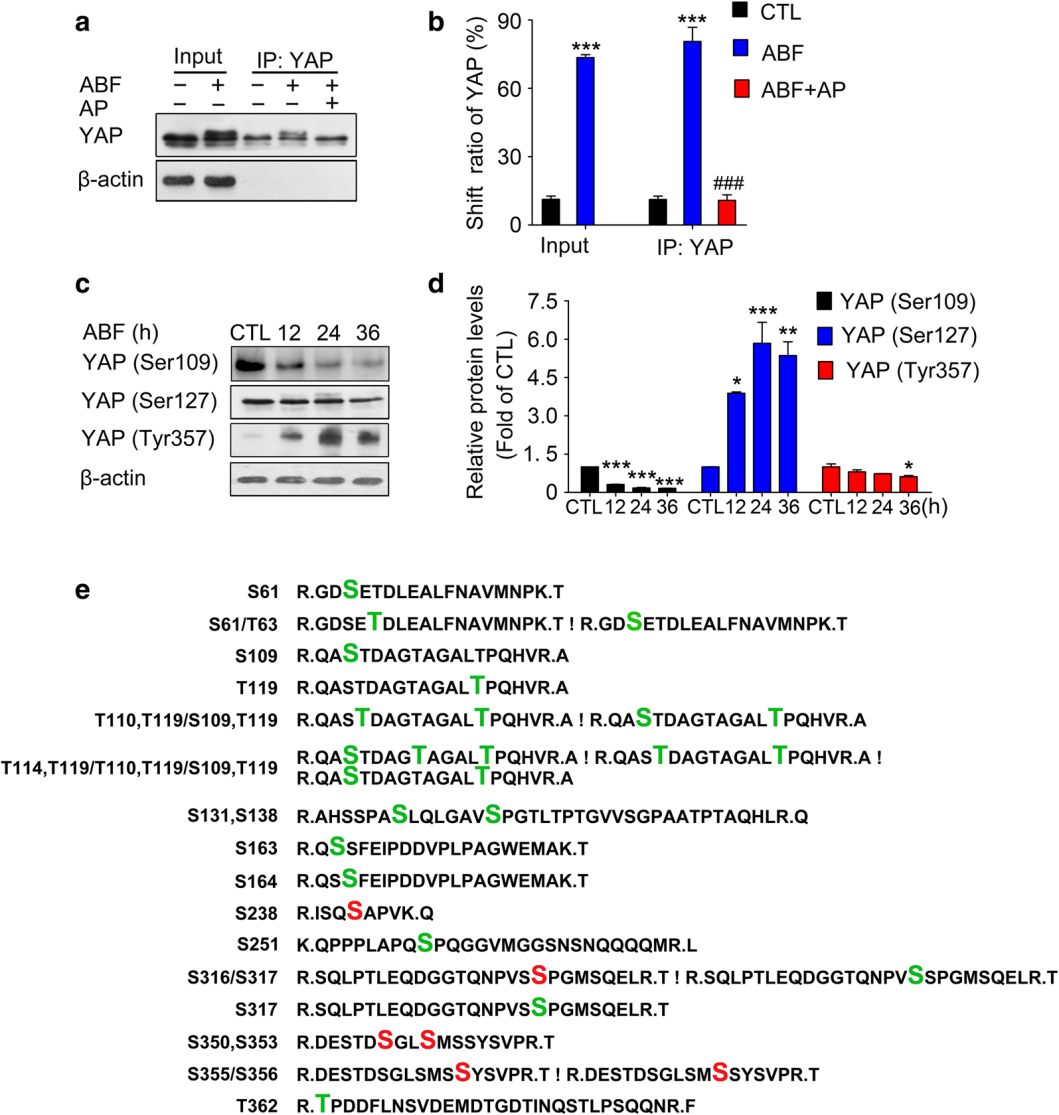
ABF induces YAP phosphorylation at multiple sites. **a** The ABF-induced shifting of YAP was caused by YAP phosphorylation. Following treatment with ABF (20 nM), cell lysates were collected and evaluated by coimmunoprecipitation and Western blotting in the presence or absence of alkaline phosphatase. **b** Quantification of the shifting of YAP, as described above. Data represent the mean ± SEM, n = 3, ^***^P < 0.001 versus the control. **c** Levels of p-YAP (Ser109), p-YAP (Ser127) and p-YAP (Try357) in MCF-7 cells treated with ABF. **d** Quantitative data of the indicated protein. Data represent the mean ± SEM, n = 3. ^*^P < 0.05, ^**^P < 0.01, ^***^P < 0.001 versus the control. **e** Identification of YAP phosphorylation sites. Following treatment, the phosphopeptides were enriched by Ti^4+^-IMAC chromatography. Potential YAP phosphopeptides were analyzed by the LC–MS/MS system. The peptide sequences of YAP recovered by mass spectrometry are labeled in black, the reported phosphorylation sites are labeled in green and the newly identified phosphorylation sites are labeled in red

It has been previously demonstrated that phosphorylation of YAP slows its electrophoretic migration [33]. To further explore the reason for the mobility shift of YAP in ABF-treated cells, YAP was immunoprecipitated and reacted with alkaline phosphatase. The slow migrating form of YAP was observed in immunoprecipitates from ABF-treated cells and, importantly, was not present in immunoprecipitates that reacted with alkaline phosphatase (Fig. 3a, b), which indicates that phosphorylation is a major event that contributes to the YAP migration shift induced by ABF. Then, we examined the levels of three major phosphorylation sites of YAP, Ser109, Ser127 and Tyr357, by Western blotting and found a significant increase in the phosphorylation level of YAP at Tyr357 (Fig. 3c, d). However, the levels of p-YAP (Ser109) and p-YAP (Ser127) were decreased (Fig. 3c, d), which indicates the presence of additional phosphorylation sites regulated by ABF. To map the potential phosphorylation sites of YAP, the phosphopeptides derived from ABF-treated cells were enriched by Ti^4+^-IMAC chromatography and further subjected to the reverse-phase nano-LC–MS/MS analysis. Mass spectrometry analysis identified 19 phosphorylation sites, with a distribution of 14 phosphoserine and 5 phosphothreonine residues (Fig. 3e). Among them, 6 new serine phosphorylation sites, Ser238, Ser316, Ser350, Ser353, Ser355 and Ser356 were identified. Together, these data indicate that the ABF-induced mobility shift of YAP is derived from its phosphorylation at multiple sites.

### ABF induces YAP phosphorylation through the JNK pathway rather than the Hippo pathway

**Fig. 4:**
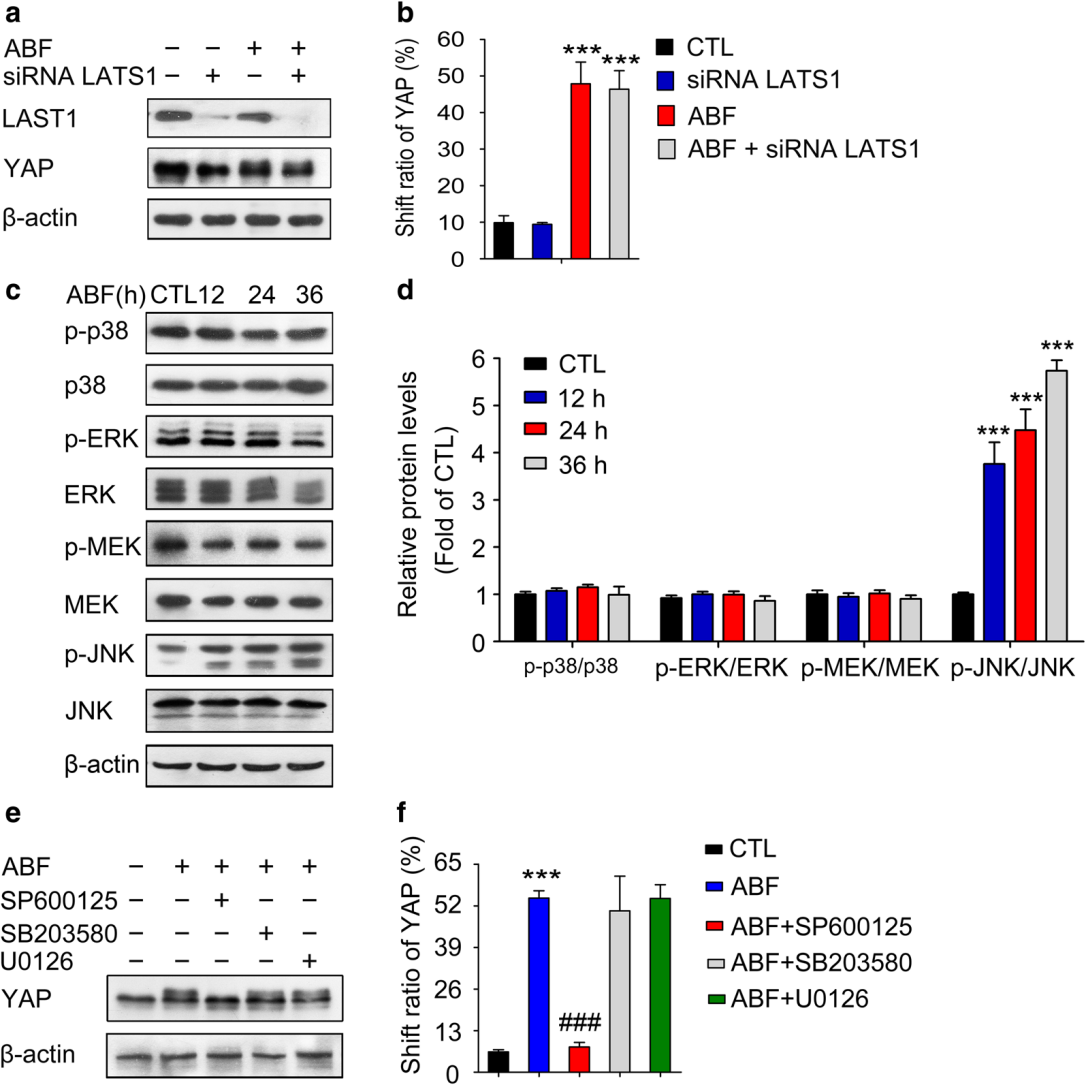
JNK signaling is involved in ABF-induced YAP phosphorylation. **a** LAST1 was not involved in YAP shift induced by ABF. Whole cell lysates were extracted from MCF-7 cells treated with ABF (20 nM) in the presence or absence of LAST1 siRNA. The lysates were evaluated by Western blotting. **b** Quantitative results for the shifting of YAP were analyzed. Data represent the mean ± SEM, n = 3. ^***^P < 0.001 versus the control. **c** ABF increased the level of p-JNK in MCF-7 cells. Lysates from cells treated with or without ABF were evaluated by Western blotting with the indicated antibodies. **d** Quantitative results of relative protein expression were analyzed. Data represent the mean ± SEM, n = 3, ^***^P < 0.001 versus the control. **e** Effects of different kinase inhibitors on YAP shifting induced by ABF. Following treatment with ABF (20 nM) in the presence or absence of SP600125, SB203580 and U0126, cell lysates were collected and subjected to Western blotting. **f** Quantification of the YAP shift was analyzed by ImageJ software. Data represent the mean ± SEM, n = 3. ^***^P < 0.001 versus the control; ^###^*P* < 0.001 versus the ABF group

It has been well established that activation of the Hippo pathway results in the phosphorylation of YAP at Ser127 [33–35]. Here, the level of YAP phosphorylation at Ser127 was slightly decreased (Fig. 3c, d), and inhibition of the Hippo pathway through siRNA targeting LATS1 had no effect on the mobility shift of YAP (Fig. 4a, b), which implies that the phosphorylation of YAP was not dependent on the classic Hippo pathway. Given that p38, ERK, MEK and JNK signaling is involved in the phosphorylation of YAP [36–38], we tested whether ABF-mediated YAP phosphorylation was mediated by activation of the above kinases. We found that ABF treatment did not lead to any obvious changes in the levels of p38, p-p38, ERK1/2, p-ERK1/2, MEK, or p-MEK (Ser217/221) but significantly increased the phosphorylation of JNK (Fig. 4c, d). Accordingly, treatment with SP600125, a JNK inhibitor, completely converted ABF induced slow-migrating bands to fast-migrating bands, whereas no such effect was induced by pretreatment of cells with SB203580 (a p38 MAPK inhibitor) or U0126 (a MEK1/2 inhibitor) (Fig. 4e, f). Thus, these data indicate that, rather than p38, ERK1/2 and MEK kinases, JNK is responsible for the YAP shift/phosphorylation induced by ABF.

### JNK-mediated multisite phosphorylation of YAP contributes to apoptosis induced by ABF

**Fig. 5:**
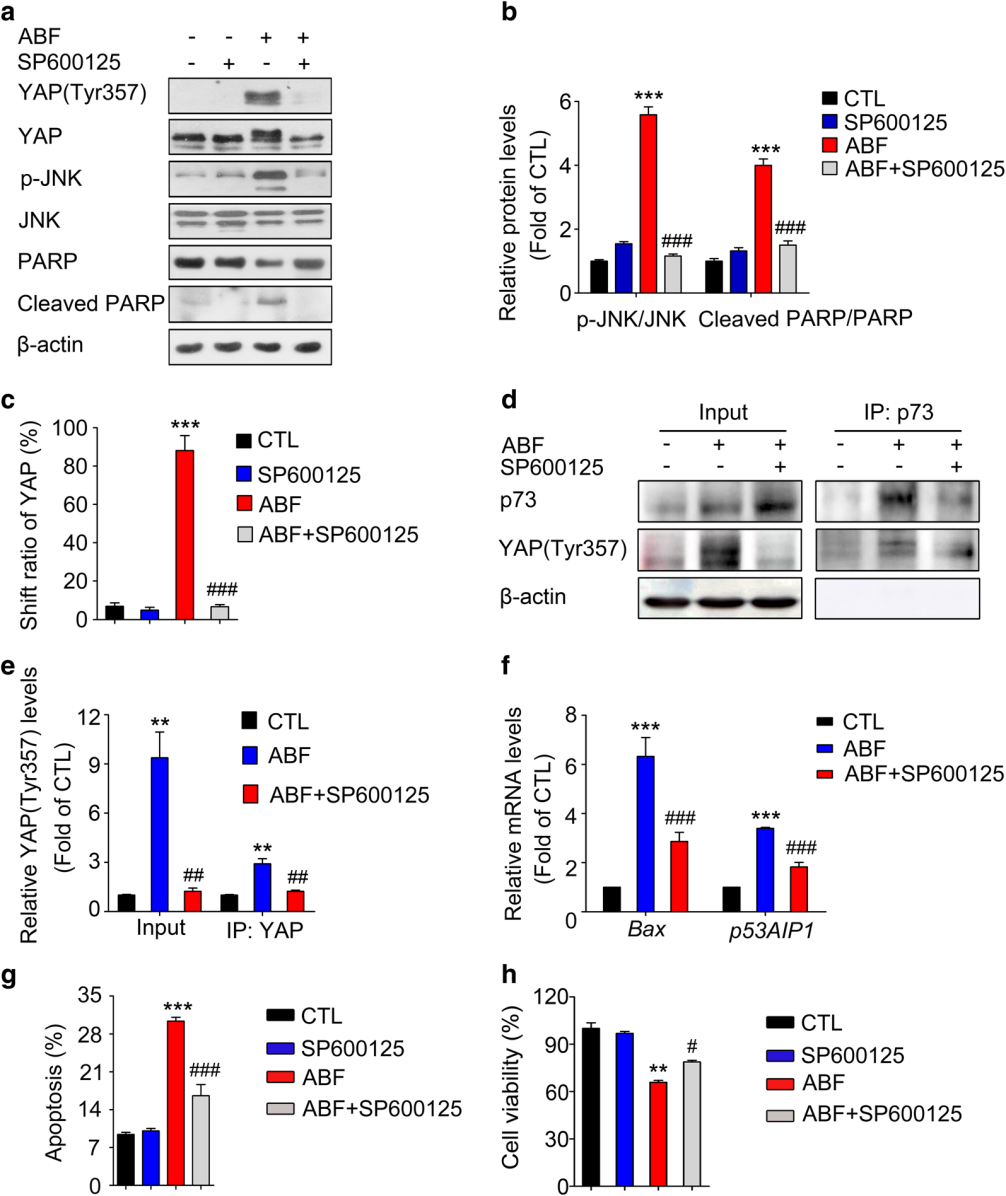
ABF-induced apoptosis requires the JNK-mediated multisite phosphorylation of YAP. **a** The JNK inhibitor SP600125 antagonized the ABF-induced multisite phosphorylation of YAP. **b**, **c** Quantitative data of the indicated proteins **b** and the YAP shift **c** were analyzed. Data represent the mean ± SEM, n = 3. ^***^P < 0.001 versus the control group; ^###^*P* < 0.001 versus the ABF group. **d** SP600125 suppressed the interaction of YAP and p73 induced by ABF. The YAP/p73 complex was pulled down by coimmunoprecipitation with an anti-p73 antibody. **e** Quantitative results of the YAP/p73 complex in ABF-treated cells. Data represent the mean ± SEM, n = 3, ^**^P < 0.01 versus the control; ^##^*P* < 0.01 versus the ABF group alone. **f** SP600125 reversed the ABF-induced upregulation of *Bax* and *p53AIP1*. The mRNA expression levels were examined by quantitative real-time PCR. Each column represents the mean ± SEM (n = 3). **g**, **h** SP600125 antagonized the ABF-induced apoptosis in MCF-7 cells. Cells were pretreated with SP600125 and then incubated with ABF. Apoptosis (**g**) and cell viability (**h**) were analyzed. Each column represents the mean ± SEM (n = 3). ^**^P < 0.01 and ^***^P < 0.001 versus the control; ^#^*P* < 0.05 and ^###^*P* < 0.001 versus the ABF group

It has been reported that phosphorylated YAP preferentially recruits pro-apoptotic promoters under apoptotic conditions [15]. We next asked whether ABF-induced JNK-mediated YAP phosphorylation contributes to the induction of apoptosis. As shown in Fig. 5a, c, SP600125 treatment completely abolished the YAP mobility shift and the phosphorylation of YAP at Tyr357 induced by ABF (Fig. 5a, c), which provides evidence that ABF induced YAP multisite phosphorylation via JNK signaling. Furthermore, the ABF-induced upregulation of cleaved PARP was dramatically attenuated by SP600125 (Fig. 5a, b), and treatment with SP600125 decreased the YAP/p73 interaction as well as the mRNA levels of ABF-induced *Bax* and *p53AIP1* (Fig. 5d–f). The addition of SP600125 also significantly attenuated the proportion of apoptotic cells (Fig. 5g) and the inhibition of cell viability (Fig. 5h) in the ABF-treated group. Taken together, these results demonstrate that ABF-induced apoptosis requires the multisite phosphorylation of YAP via the JNK signaling pathway.

## Discussion

Increasing numbers of studies have been performed to identify the importance of bufadienolides as anticancer agents. However, the mechanisms by which bufadienolides induce apoptosis in breast cancer cells have not been defined. Here, we reveal a novel mechanism of ABF, a representative bufadienolide, in the induction of MCF-7 cell apoptosis through JNK-mediated YAP phosphorylation. Our study also indicates that ABF can be developed as a novel and potential therapeutic agent for breast carcinomas.

Previous reports have shown that bufalin enhanced TRAIL-induced apoptosis in MCF-7 and MDA-MB-231 breast cancer cells by activating the extrinsic apoptotic pathway [39], the downregulation of Cbl [40] and the inhibition of STAT3/Mcl-1 [41]. As an inhibitor of SRC-3, bufalin reduced tumor growth in an MDA-MB-231-LM3.3 xenograft tumor model. Yuan et al. [42] have found that ABF inhibits the proliferation and survival of HER2-overexpressing breast cancer MCF-7/HER2 and T47D/HER2 cells through the inhibition of AKT, ERK and E2F1. Instead of these reported mechanisms, we find that ABF-induced phosphorylation of YAP through JNK signaling enhances the p73-mediated transcription of *Bax* and *p53AIP1*, and subsequently induces apoptosis in MCF-7 cells. However, this phenomenon was not observed in two types of highly invasive breast cancer cell lines, MDA-MB-231 and MDA-MB-435 (data not shown). MDA-MB-231 and MDA-MB-435 cells are ER-negative, PR-negative and HER2-negative, but MCF-7 cells are ER-positive and PR-positive [43]. Considering that YAP enhances ER transactivation via WW domain binding protein-2 (WBP-2) [44] and that decreased expression of YAP is associated with ER and PR negativity in patients with invasive breast cancer [45], we speculate that YAP motility shift and the multisite phosphorylation of YAP induced by ABF only occur in MCF-7 cells, which are ER- and PR-positive. The exact connections between ER/PR and ABF-induced YAP phosphorylation will be further investigated. Moreover, the YAP-associated anti-cancer activities induced by ABF will be defined using in vivo experiments in the future.

To date, the phosphorylation of YAP under different conditions is widely observed to determine YAP function, including its oncogenic and pro-apoptotic activities [7]. The phosphorylation of YAP at Ser94 promotes the interaction between YAP and TEAD, which is required for YAP-induced cell proliferation. Additionally, the YAP-S94A mutation abolishes YAP-TEAD binding, EMT and oncogenic transformation [12, 13]. In the canonical Hippo pathway, Ser127 and Ser381 are the principal sites of YAP phosphorylation, which induce YAP degradation and suppress its oncogenic activity [14]. Under pro-apoptotic conditions, such as cisplatin and UV treatment, YAP is phosphorylated at the Tyr357 residue, which enhances the transcription of pro-apoptotic genes, such as *Bax*, *p53AIP1*, *PML*, and *puma*; this supports its function as a tumor suppressor [15, 30, 32, 46, 47]. Previously, we found that ABF led to double-strand DNA breaks and triggered the DNA damage response [22]. We show here that the phosphorylation of YAP can be induced by ABF, which triggered apoptosis along with the nuclear translocation of YAP. Phosphorylation of YAP also increased the mRNA levels of *Bax* and *p53AIP1*, which is consistent with the results of previous studies. Hence, our work provides a useful case for understanding YAP phosphorylation as a potential key element in the regulation of the pro-apoptotic properties of YAP in DNA damage-induced apoptosis.

It is well known that the Hippo pathway regulates YAP, and several recent studies have also demonstrated that YAP is regulated by some Hippo-independent mechanisms, such as CK1 [14], the JNK pathway [38], PKC [48], MAPK signaling [49], and cyclin-dependent kinases (CDKs) [50]. Here, the mass spectrometry analysis revealed 19 potential phosphorylation sites on YAP. Overall, we speculated as to the existence of the following: 5 potential sites, Ser61, Thr63, Ser109, Ser138 and Ser164, phosphorylated by CK1 [14]; 2 potential sites, Ser61 and Thr119, phosphorylated by AMPK [49]; and 5 potential serine sites, Ser61, Ser109, Ser131, Ser163 and Ser164, phosphorylated by the Hippo pathway [14, 33, 35]; 5 potential sites, Thr119, Ser138, Ser251, Ser317 and Thr362, phosphorylated by CDK1 [50, 51]; and 5 potential sites, Ser109, Thr110, Thr119, Ser163, and Ser164 phosphorylated by PKC [48]. Recently, Tomlinson et al. reported that the phosphorylation of YAP at Thr119, Ser138, Thr154, Ser317 and Thr362 by JNK signaling promotes UV-induced apoptosis in HaCaT cells [38]. In the present study, we also identified most of the reported phosphorylation sites (Thr119, Ser138, Ser317 and Thr362) and confirmed the JNK-induced YAP phosphorylation at multiple sites by ABF. Additionally, the slow-migrating form of YAP was also observed in both nuclear and cytoplasmic fractions (Additional file 1: Fig. S2), which is consistent with the total protein, and further confirmed the phosphorylation of YAP at multiple sites. Nevertheless, it is still possible that additional mechanisms of YAP phosphorylation in response to ABF treatment exist, as indicated by 6 novel serine residues (Ser238, Ser316, Ser350, Ser353, Ser355 and Ser356) that are phosphorylated by ABF. Although the functions of these sites are unknown, they may be a sign of the pro-apoptotic role of YAP in ABF-treated MCF-7 cells.

## Conclusions

In summary, this study reveals a new molecular mechanism of ABF-induced apoptosis by the JNK-mediated multisite phosphorylation of YAP and presents experimental data for expanding the specific functions of YAP in the induction of cell apoptosis and for identifying a potential therapeutic agent for ER-positive breast cancer patients.

## Additional file

**Additional file 1: Table S1.** The sequence-specific oligonucleotide primers. **Table S2.** The target siRNA duplexes. **Figure S1.** Representative data from the Annexin V/PI apoptosis assay. Following ABF treatment, MCF-7 cells were subjected to Annexin V/PI staining and analyzed by flow cytometry, n = 3. **Figure S2.** Distribution of YAP in the nuclei and cytoplasm of ABF-treated MCF-7 cells. Nuclear and cytoplasmic fractions were collected and evaluated by Western blotting. Lamin B1 was used as the nuclear protein control, while GAPDH was used as the cytoplasmic protein control.

## Abbreviations

YAP: yes-associated protein
ABF: arenobufagin
TCMs: traditional Chinese medicines
PFA: paraformaldehyde
DAPI: 4′,6-Diamidino-2-phenylindole
MTT: 3-(4,5-dimethylthiazol-2-yl)-2,5-diphenyltetrazolium bromide
EMT: epithelial-to-mesenchymal transition
PVDF: polyvinylidene
CDK: cyclin-dependent kinase
