# Supplementary material for: Arenobufagin induces MCF-7 cell apoptosis by promoting JNK-mediated multisite phosphorylation of Yes-associated protein
Source: Cancer Cell Int. 2018 Dec 18;18:209. doi: 10.1186/s12935-018-0706-9 (PMC6299615; doi:10.1186/s12935-018-0706-9)
Supplement: Supplementary file 1 — Additional file 1: Table S1. The sequence-specific oligonucleotide primers. Table S2. The target siRNA duplexes. Figure S1. Representative data from the Annexin V/PI apoptosis assay. Following ABF treatment, MCF-7 cells were subjected to Annexin V/PI staining and analyzed by flow cytometry, n = 3. Figure S2. Distribution of YAP in the nuclei and cytoplasm of ABF-treated MCF-7 cells. Nuclear and cytoplasmic fractions were collected and evaluated by Western blotting. Lamin B1 was used as the nuclear protein control, while GAPDH was used as the cytoplasmic protein control. [file 12935_2018_706_MOESM1_ESM.docx]

**Additional file**

**Arenobufagin induces MCF-7 cell apoptosis by promoting JNK-mediated multisite phosphorylation of Yes-associated protein**

**Running title: ABF induces apoptosis through the phosphorylation of YAP**

Li-Juan Deng^1,2^^,†^, Ming Qi^1,3†^, Qun-Long Peng^1,3^, Min-Feng Chen^1,3^, Qi Qi^4^, Jia-Yan Zhang^1,3^, Nan Yao^1,3^, Mao-Hua Huang^1,3^, Xiao-Bo Li^1,3^, Yin-Hui Peng^1,3^, Jun-Shan Liu^5^, Deng-Rui Fu^6^, Jia-Xu Chen^2^, Wen-Cai Ye^1,3,*^, Dong-Mei Zhang^1,3,*^

^*^Correspondence: dmzhang701@jnu.edu.cn

^†^ Li-Juan Deng and Ming Qi contributed equally to the work;

**Contents**

Title page… …………………….……………………………………….…….……..1

Table S1 The sequence-specific oligonucleotide primers…….………………………2

Table S2 The target siRNA duplexes…………………………………………………2

Figure S1 Representative data from the Annexin V/PI apoptosis assay…....2

Figure S2 Distribution of YAP in the nuclei and cytoplasm fractions in ABF-treated MCF-7 cells……..…………………………………………………....…......3

Table S1. The sequence-specific oligonucleotide primers

| Target genes | Primers |
| --- | --- |
| *BAX* | forward: 5’-AGGATGCGTCCACCAAGAAG-3’; |
|  | reverse: 5’-AGCTGCCACTCGGAAAAAGA-3’. |
| *p53AIPI* | forward: 5’-CCGGGCCAGGAGTAAGTAAC-3’; |
|  | reverse: 5’-ACAACGCCGAAGAGGATCAG-3’. |
| *GAPDH* | forward: 5’-CCACCCCAATGTCTCTGTT-3’; |
|  | reverse: 5’-CAACCTGGTGCTCCGTGTAT-3’. |

Table S2. The target siRNA duplexes

| Target genes | siRNA duplex sequences |
| --- | --- |
| *YAP* | forward: 5’-UCUGCAGUUGGGAGCUGUUTT-3’; |
|  | reverse: 5’-AACAGCUCCCAACUGCCAGATT-3’. |
| *LATS1* | forward: 5’-GGAGUGUUACUCCUCCACCTT-3’; |
|  | reverse: 5’-GGUGGAGGAGUAACACUCCTT-3’. |
| Negative siRNA duplex | forward: 5’-UUCUCCGAACGUGUCACGUTT-3’; |
|  | reverse: 5’-ACGUGACACGUUCGGAGAATT-3’. |


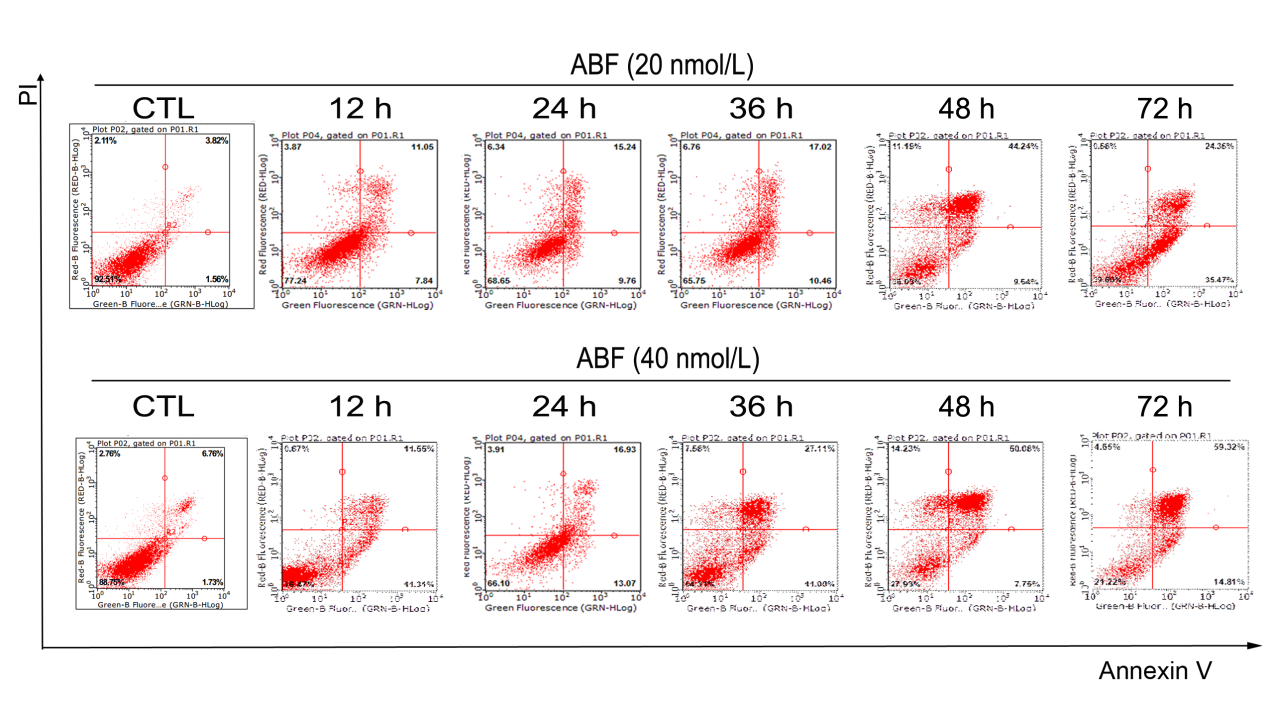


**Figure S1** Representative data from the Annexin V/PI apoptosis assay. Following ABF treatment, MCF-7 cells were subjected to Annexin V/PI staining and analyzed by flow cytometry, n = 3.


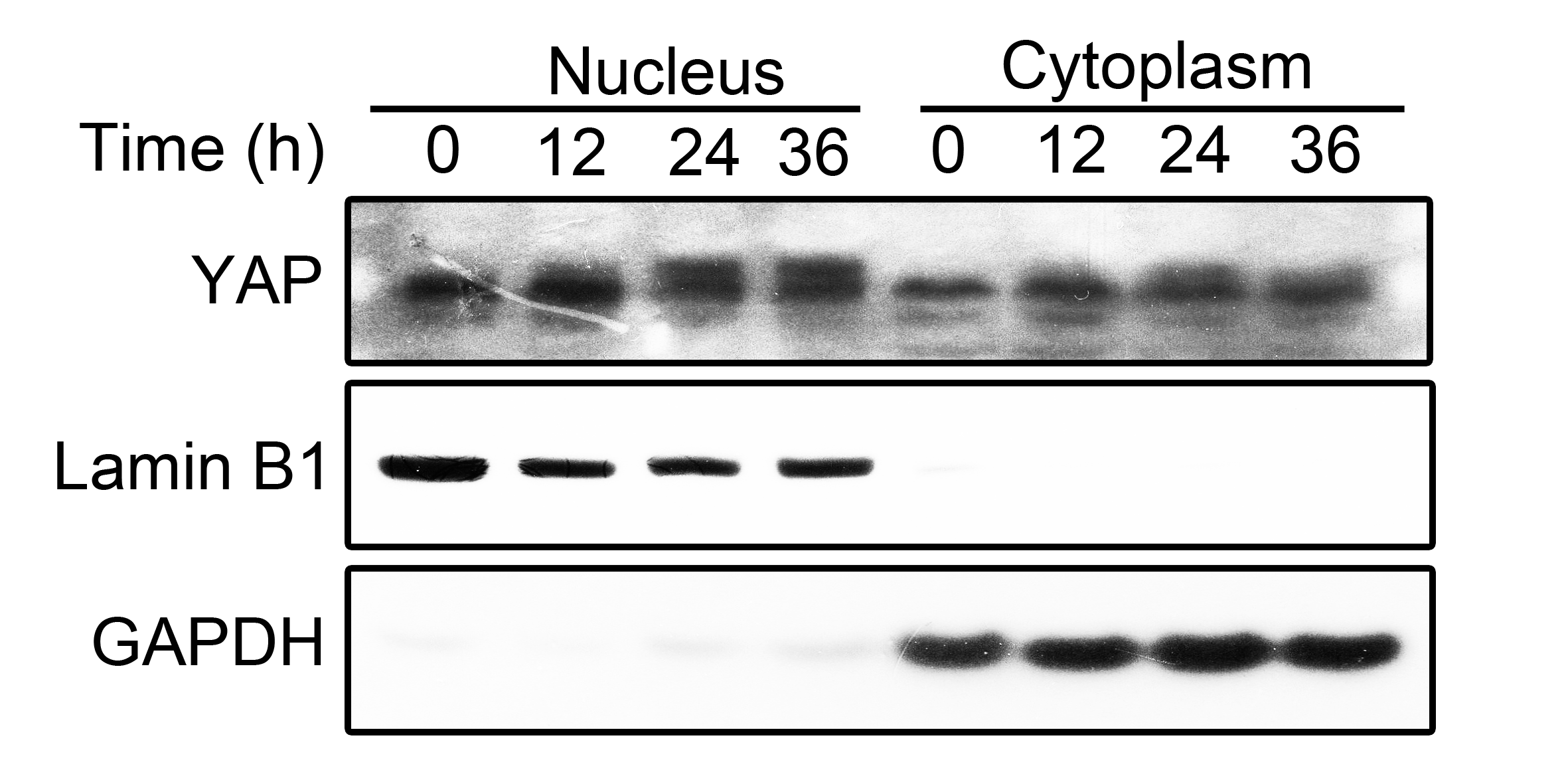


**Figure S2** Distribution of YAP in the nuclei and cytoplasm of ABF-treated MCF-7 cells. Nuclear and cytoplasmic fractions were collected and evaluated by Western blotting. Lamin B1 was used as the nuclear protein control, while GAPDH was used as the cytoplasmic protein control.
